# Complete conversion of all typical glycosylated protopanaxatriol ginsenosides to aglycon protopanaxatriol by combined bacterial β-glycosidases

**DOI:** 10.1186/s13568-018-0543-1

**Published:** 2018-01-24

**Authors:** Eun-Joo Yang, Tae-Hun Kim, Kyung-Chul Shin, Deok-Kun Oh

**Affiliations:** Department of Bioscience and Biotechnology, Konkuk University, Seoul, 05029 Republic of Korea

**Keywords:** Aglycon protopanaxatriol, β-Glycosidase, *Dictyoglomus turgidum*, *Pyrococcus furiosus*, *Panax notoginseng* root extract

## Abstract

Aglycon protopanaxatriol (APPT) has valuable pharmacological effects such as anti-inflammatory and anti-stress activities. However, the complete conversion of all typical glycosylated protopanaxatriol ginsenosides to APPT has not been achieved to date. β-Glycosidase from the hyperthermophilic bacterium *Dictyoglomus turgidum* (DT-bgl) hydrolyzes the glucose residues at C-6 and the inner glucose at C-20 in protopanaxatriol (PPT), but not the outer rhamnose residues at C-6. In contrast, β-glycosidase from the hyperthermophilic bacterium *Pyrococcus furiosus* (PF-bgl) hydrolyzes the outer rhamnose residue at C-6 but not the inner glucose residues at C-6 and C-20 in PPT. Thus, the combined use of DT-bgl and PF-bgl resulted in the complete the conversion of all typical glycosylated PPT ginsenosides, including R1, R2, Re, Rg1, Rg2, Rh1, Rf, F1, F3, and F5, to APPT. DT-bgl combined with PF-bgl completely hydrolyzed 1.0 mg ml^−1^ R1 and 1.0 mg ml^−1^ total PPT-type ginsenosides in *Panax notoginseng* root extract to 0.5 and 0.63 mg ml^−1^ APPT for 4 and 3 h, with molar conversions of 100% and productivities of 125 and 210 mg l^−1^ h^−1^, respectively. To the best of our knowledge, this is the first report of the complete conversion of all typical glycosylated PPT ginsenosides to APPT and the highest productivity of APPT obtained from ginseng extract achieved to date.

**Electronic supplementary material:**

The online version of this article (10.1186/s13568-018-0543-1) contains supplementary material, which is available to authorized users.

## Introduction

Ginseng has been used as a traditional medicine in Asian countries because it has bioactive ginsenosides. Ginsenosides are the main substances in ginseng with pharmacological effects and they are mainly divided into protopanaxatriol (PPT)-type and protopanaxadiol (PPD)-type ginsenosides according to the number and position of the hydroxyl groups. PPT-type ginsenosides contain different sugars at C-6 and C-20 linked to the aglycon PPT (APPT) skeleton. The outer sugar at C-6 in PPT-type ginsenosides is glucose, rhamnose, or xylose, whereas the inner sugars at C-6 and C-20 are always glucose (Shin and Oh 2016). The outer sugar at C-20 in ginsenoside F3 or F5 is arabinopyranose or arabinofuranose, respectively. Typical PPT ginsenosides consist of R1, R2, Re, Rg1, Rg2, Rh1, Rf, F1, F3, F5, and APPT according to the presence or absence of different types of sugars (Yang et al. 2014). The composition and types of PPT ginsenosides in ginseng extract are different depending on the parts and species of ginseng. The types of PPT ginsenosides in the root, leaf, and berry of *Panax ginseng* or *Panax quinquefolius* are Re, Rg1, Rg2, and Rh1. *P. ginseng* flower buds include F1, F3, and F5, and *Panax notoginseng* root contains R1, Rg1, and Re. In particular, R1 only exists in *P. notoginseng* root (Li et al. 2016a; Wang et al. 2009a; Xie et al. 2004).

The pharmacological effects of PPT-type ginsenosides are as follows: Rg1 and Rb1 have anti-aging and anti-amnestic activities (Cheng et al. 2005), ginsenoside Re exhibits anti-diabetic activity (Xie et al. 2005), F1 protects keratinocytes from ultraviolet B-induced apoptosis (Lee et al. 2003), Rg1 and Rg2 protect against Alzheimer disease (Li et al. 2016b), and APPT has anti-fatigue, anti-inflammatory, anti-stress, and memory enhancement effects, and inhibits lipogenesis and adipocyte differentiation (Lee et al. 2015; Oh et al. 2015a, b; Wang et al. 2009b; Zhang et al. 2014). Minor ginsenosides with one sugar or no sugars are more rapidly absorbed into the bloodstream and have better pharmacological effects than major ginsenosides with three or four sugars (Kim et al. 2005). Therefore, specific sugar-hydrolysis is required to obtain biologically and pharmacologically active PPT-type ginsenosides.

APPT is a final metabolite of PPT-type ginsenosides by human intestinal bacteria that is not present in nature (Bae et al. 2005). The production of APPT is relatively difficult because it requires the complete hydrolysis of all the sugar moieties linked to the skeleton of PPT-type ginsenosides. Although APPT is currently produced via alkaline hydrolysis, alkaline treatment cannot exceed a yield of 80%, shows low productivity, and produces a variety of by-products (Cui et al. 1993). In contrast, enzymatic hydrolysis can hydrolyze specific sugar moieties with high productivity and no by-products. Recently, β-glycosidase from *Dictyoglomus turgidum* (DT-bgl) was reported to be the most efficient APPT producer. However, this enzyme cannot hydrolyze the outer rhamnose residue at C-6 in PPT (Lee et al. 2014). Thus, DT-bgl does not produce APPT from Re, Rg2, and F3.

In the present study, to convert all typical glycosylated PPT ginsenosides to APPT, β-glycosidase from *Pyrococcus furiosus* (PF-bgl), which can hydrolyze the outer rhamnose residues at C-6 (Oh et al. 2014), was added to DT-bgl. The combination of DT-bgl with PF-bgl resulted in the complete conversion of all typical glycosylated PPT ginsenosides to APPT with a molar conversion of 100%.

## Materials and methods

### Materials

The ginsenoside standards R1 and R2 were purchased from Ambo Laboratories (Daejeon, Republic of Korea), and Rg1, Rh1, APPT, F1, F3, and F5 standards were obtained from Star Ocean Ginseng (Changshu, Suzhou, China).

### Preparation of notoginseng root extract

For the extraction of ginsenosides, 20 g of dried notoginseng root powder was suspended in 200 ml of 80% (v/v) methanol and incubated at 80 °C for 15 h. To remove solid, the mixed solution was filtered through a 0.45 μm filter and the methanol in the filtrate was eliminated by evaporation. Distilled water at 200 ml was added to the methanol-free residue. The dissolved extract was applied to a packed column containing 50 ml Diaion HP-20 resin to attach the ginsenosides to the resin. The hydrophilic compounds including unbound sugars in the resin was removed by eluting with 200 ml distilled water, and the ginsenosides attached to the resin were then obtained by eluting with 80% (v/v) methanol. The methanol in the eluent was eliminated by evaporation, and the methanol-free residue was dissolved in 200 ml distilled water. The resulting solution was used as the notoginseng root extract.

### Culture conditions and enzyme preparation

Genes of β-glycosidases from *D. turgidum* DSM 6724 (DSMZ, Brauschweig, Germany) and *P. furiosus* DSMZ 3638 were cloned as described previously (Lee et al. 2014; Oh et al. 2014). Recombinant *Escherichia coli* ER2566 (New England Biolabs, Herfordshire, UK) expressing DT-bgl (GenBank Accession Number YP_002352162) or PF-bgl (GenBank Accession Number AAC25555) was cultivated at 37 °C in a 2000 ml flask containing 500 ml Luria–Bertani medium mixed with 20 µg ml^−1^ kanamycin with rotation at 200 rpm in a shaker. When the optical density of the culture broth at 600 nm reached 0.6, 0.1 mM isopropyl-β-d-thiogalactopyranoside was added to the broth, followed by incubation at 16 °C for a further 16 h with rotation at 150 rpm to induce β-glycosidase expression. The induced recombinant cells were obtained from the culture broth by centrifugation at 12,000×*g* at 4 °C for 30 min, suspended in 50 mM citrate/phosphate buffer (pH 6.0), and lysed by sonication on ice. The sonicated cells were eliminated by centrifugation at 12,000×*g* at 4 °C for 30 min, and the obtained supernatant was filtered through a 0.45 μm filter. The filtrate was heated at 75 °C for 30 min and centrifuged at 12,000×*g* for 30 min to precipitate and remove the insoluble proteins of the heat-treated suspension. The soluble protein as the supernatant was used as the enzyme for the biotransformation of ginsenosides.

### Hydrolytic activity and productivity

One unit (U) of DT-bgl or PF-bgl activity was defined as the amount of enzyme required to liberate 1 µmol *p*-nitrophenol (*p*NP) from *p*NP-β-d-glucopyranoside per min at 80 °C and pH 6.0. The substrate specificity was determined after incubation at 80 °C for 10 min in 50 mM citrate/phosphate buffer (pH 6.0) containing 0.4 mg ml^−1^ PPT-type ginsenoside (R1, R2, Re, Rg1, Rg2, Rf, Rh1, F1, F3, or F5), DT-bgl or PF-bgl, and 4% (v/v) dimethyl sulfoxide. The DT-bgl concentration was 1.0 mg ml^−1^ for R1 and R2; 0.1 mg ml^−1^ for Re, Rf, and F_1_; and 0.5 mg ml^−1^ for Rh_1_ and Rg_1_. The PF-bgl concentration was 0.4 mg ml^−1^ for R2; 0.25 mg ml^−1^ for F3; 0.2 mg ml^−1^ for R1; 0.1 mg ml^−1^ for Re and Rg_2_; and 0.3 mg ml^−1^ for Rf. The specific activity was calculated from the decreased rate of concentration of the substrate PPT-type ginsenoside.

The time-course reactions for the biotransformation of glycosylated PPT-type ginsenoside to APPT by DT-bgl combined with PF-bgl were carried out with 1.0 mg ml^−1^ total PPT-type ginsenosides, 4.0 mg ml^−1^ DT-bgl, and 5 µg ml^−1^ PF-bgl. Productivity was calculated as the concentration of APPT per the minimal reaction time for the complete conversion to APPT.

### Optimization of reaction conditions for APPT production by DT-bgl

All reactions were performed at 80 °C in 50 mM citrate/phosphate buffer (pH 6.0). To determine the optimal concentrations of DT-bgl as an enzyme and ginsenoside R1 as a substrate, enzyme and substrate concentrations were varied from 1.0 to 8.0 mg ml^−1^ at 1.0 mg ml^−1^ R1 and from 0.5 to 2.0 mg ml^−1^ at 4.0 mg ml^−1^ DT-bgl, respectively. After 1.5 h, the concentrations of ginsenosides were determined. The time-course reactions for the biotransformation of ginsenoside R1 to APPT were carried out with 1.0 mg ml^−1^ R1 and 4.0 mg ml^−1^ DT-bgl for 8 h. To determine the optimal concentrations of DT-bgl as an enzyme and notoginseng root extract as a substrate, the DT-bgl concentration was varied from 1.0 to 8.0 mg ml^−1^ at 1.0 mg ml^−1^ total PPT-type ginsenosides in the notoginseng root extract and the concentration of notoginseng root extract was varied from 0.5 to 2.0 mg ml^−1^ at 5.0 mg ml^−1^ DT-bgl. After 5 h, the concentrations of ginsenosides were determined. The time-course reactions for the biotransformation of glycosylated PPT-type ginsenosides in notoginseng root extract to APPT were carried out with 1.0 mg ml^−1^ total PPT-type ginsenosides and 5.0 mg ml^−1^ DT-bgl for 5 h.

### Determination of the minimal concentration of PF-bgl added to DT-bgl

The minimal concentration of PF-bgl for the complete conversion of ginsenoside R1 to APPT was determined by varying the enzyme concentration from 0.156 to 10 µg ml^−1^ with 1.0 mg ml^−1^ R1 and 4.0 mg ml^−1^ DT-bgl for 6 h. The minimal added concentration of PF-bgl to DT-bgl for achieving the complete conversion of glycosylated PPT-type ginsenosides in notoginseng root to APPT was determined by varying the PF-bgl concentration from 0.025 to 0.8 mg ml^−1^ with 1.0 mg ml^−1^ total PPT-type ginsenosides in notoginseng root extract and 5.0 mg ml^−1^ DT-bgl for 4 h.

### APPT production by DT-bgl combined with PF-bgl under the optimized conditions

The time-course reactions for the biotransformation of ginsenoside R1 to APPT by DT-bgl combined with PF-bgl were carried out with 1.0 mg ml^−1^ R1, 4.0 mg ml^−1^ DT-bgl, and 5 µg ml^−1^ PF-bgl for 5 h. The same reaction conditions were used for the time-course reactions of ginsenosides F3 and F5 to APPT for 8 h. The time-course reactions for the biotransformation of glycosylated PPT-type ginsenosides in notoginseng root extract to APPT were performed with 1.0 mg ml^−1^ total PPT-type ginsenosides in notoginseng root extract, 5.0 mg ml^−1^ DT-bgl, and 0.4 mg ml^−1^ PF-bgl in 50 mM citrate/phosphate buffer (pH 6.0) at 80 °C for 4 h.

### Analytical methods

The same volume of butanol containing digoxin as an internal standard was mixed to the reaction solution. The butanol layer of the mixture was dried by evaporation, and methanol was added to the dried extract. A high-performance liquid chromatography (HPLC) system (Agilent 1100; Santa Clara, CA, USA) with a C18 column and an ultraviolet detector set to detect at 203 nm was used to determine the concentrations of ginsenosides. The absorbed ginsenosides in the C18 column (YMC, Kyoto, Japan) were eluted at 40 °C by pumping with a linear gradient of acetonitrile and water from 30:70 to 35:65 (v/v) for 17.8 min at a flow rate of 0.25 ml min^−1^ and from 35:65 to 34:66 for 19.5 min at a flow rate of 0.2 ml min^−1^ and from 34:66 to 90:10 for 24.5 min at a flow rate of 1.0 ml min^−1^.

## Results

### Substrate specificity and hydrolytic pathways of DT-bgl and PF-bgl for all typical glycosylated PPT ginsenosides

**Fig. 1:**
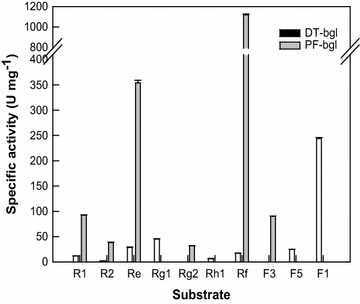
Specific activities of DT-bgl and PF-bgl for all typical PPT ginsenosides. Black and gray bar represent DT-bgl and PF-bgl, respectively

The specific activities of DT-bgl and PF-bgl for all typical glycosylated PPT ginsenosides as substrates are presented in Fig. 1. DT-bgl produced APPT but PF-bgl did not. PF-bgl had activity for Rg2 and F3 but DT-bgl did not. In contrast, DT-bgl had activity for Rg1, Rh1, F1, F5 but PF-bgl did not. The specific activity of PF-bgl for the common substrates, including ginsenosides R1, R2, Re, and Rf, were 7.8-, 19.3-, 6.0-, and 65-fold higher than those of DT-bgl, respectively. DT-bgl converted R1 and Re to R2 and Rg2, respectively, whereas PF-bgl converted both of these ginsenosides to Rg1. In particular, DT-bgl showed the lowest activity for R2 among the PPT-type ginsenosides, indicating that the limiting step for APPT production is the conversion of R2 to Rh1.

**Fig. 2:**
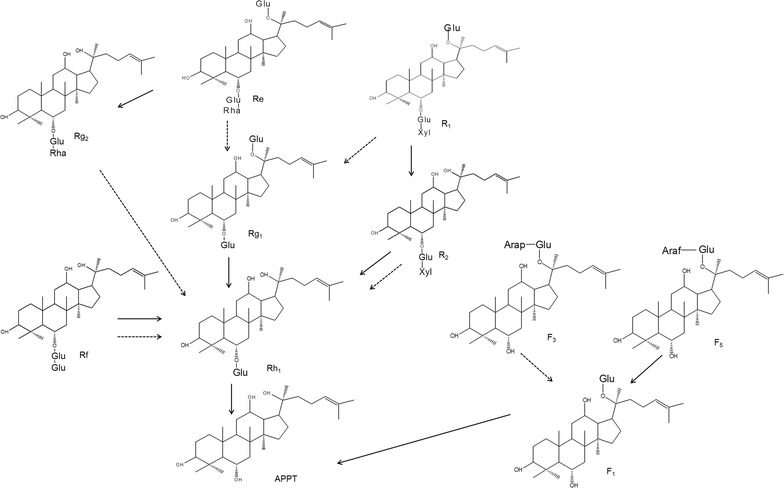
Hydrolytic pathways of glycosylated protopanaxatriol (PPT) ginsenosides to aglycon protopanaxatriol (APPT) catalyzed by β-glycosidases from *D. turgidum* (DT-bgl) and *P. furiosus* (PF-bgl). Solid and dotted lines represent DT-bgl and PF-bgl, respectively

DT-bgl had the following hydrolytic pathways of glycosylated PPT-type ginsenosides: R1 → R2 → Rh1 → APPT, Rg1/Rf → Rh1 → APPT, F5 → F1 → APPT, and Re → Rg2. DT-bgl could not hydrolyze the outer sugar rhamnose residue at C-6 in ginsenosides Re and Rg2 and the outer sugar arabinopyranose residue at C-20 in ginsenoside F3. Since PF-bgl could hydrolyze the rhamnose at C-6 in PPT-type ginsenosides and the arabinopyranose at C-20, PF-bgl converted these ginsenosides to Rg1, Rh1, and F1, which were transformed to APPT by DT-bgl. Thus, DT-bgl combined with PF-bgl converted all typical glycosylated PPT ginsenosides to APPT via the hydrolytic pathways of R1 → R2/Rg1 → Rh1 → APPT, Re → Rg1/Rg2 → Rh1 → APPT, Rf → Rh1 → APPT, and F5/F3 → F1 → APPT (Fig. 2).

### Conversion of ginsenoside R1 and glycosylated PPT-type ginsenosides in notoginseng root extract to APPT by DT-bgl under the optimized conditions

**Fig. 3:**
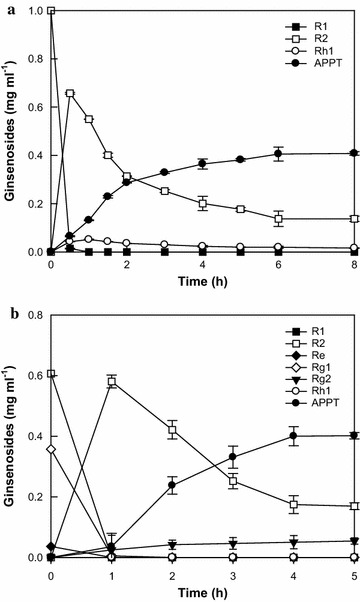
Time-course reactions for the biotransformations of PPT-type ginsenosides to APPT by DT-bgl. **a** Ginsenoside R1. The reactions were performed at 80 °C in 50 mM citrate/phosphate buffer (pH 6.0) containing 1.0 mg ml^−1^ R1 and 4.0 mg ml^−1^ DT-bgl for 8 h. **b** PPT-type ginsenosides in notoginseng root extract. The reactions were performed at 80 °C in 50 mM citrate/phosphate buffer (pH 6.0) containing 1.0 mg ml^−1^ total PPT-type ginsenosides and 5.0 mg ml^−1^ DT-bgl for 5 h. Data represent the means of three experiments, and error bars represent the standard deviation

The optimal temperature and pH for APPT production from R1 using DT-bgl were previously determined to be 80 °C and 6.0, respectively (Lee et al. 2014). APPT production was investigated at enzyme concentrations ranging from 1.0 to 8.0 mg ml^−1^ at 1.0 mg ml^−1^ R1 as a substrate for 1.5 h (Additional file 1: Figure S1a). APPT production from R1 increased with increasing DT-bgl concentration up to 4.0 mg ml^−1^ and reached a plateau at concentrations above 4.0 mg ml^−1^. APPT production was tested by varying the concentration R1 ranging from 0.5 to 2.0 mg ml^−1^ at 4.0 mg ml^−1^ enzyme for 1.5 h (Additional file 1: Figure S1b). APPT production increased with increasing R1 concentration up to 1.0 mg ml^−1^ and reached a plateau above this level. Thus, the optimal concentrations of DT-bgl and R1 were determined to be 4.0 and 1.0 mg ml^−1^, respectively. Under the optimized conditions, the time-course reactions for the biotransformation of R1 to APPT were performed with 1.0 mg ml^−1^ R1 and 4.0 mg ml^−1^ DT-bgl for 8 h (Fig. 3a). After 6 h, DT-bgl produced 0.40 mg ml^−1^ APPT, with a productivity of 67 mg l^−1^ h^−1^ and a molar conversion of 80.6%, and also produced 0.14 mg ml^−1^ R2, with a molar conversion of 16.9%. The intermediate R2 was not decreased after 6 h due to the significantly low activity of DT-bgl for R2.

Notoginseng root extract containing 3.78 mg ml^−1^ total PPT-type ginsenosides was obtained by extraction with 80% methanol. The content of specific PPT-type ginsenosides among total PPT-type ginsenosides in notoginseng root extract followed the order R1 (54.7%, w/w) > Rg1 (37.7%) > Re (7.6%), indicating that efficient hydrolysis of ginsenoside R1 is essential for the increased biotransformation of glycosylated PPT-type ginsenosides in notoginseng root extract to APPT. APPT production was investigated at enzyme concentrations ranging from 1.0 to 8.0 mg ml^−1^ at 1.0 mg ml^−1^ total PPT-type ginsenosides in notoginseng root extract as a substrate for 5 h (Additional file 1: Figure S2a). APPT production from PPT-type ginsenosides in ginseng root extract increased proportionally with enzyme concentration up to 5.0 mg ml^−1^ and then reached a plateau at higher concentrations. APPT production was tested by varying the R1 concentration in notoginseng root extract from 0.5 to 2.0 mg ml^−1^ at 5.0 mg ml^−1^ enzyme for 5 h (Additional file 1: Figure S2b). APPT production was maximal at 1.0 mg ml^−1^ total PPT-type ginsenosides in notoginseng root extract. Thus, the optimal concentrations of DT-bgl and R1 in notoginseng root extract were determined to be 5.0 and 1.0 mg ml^−1^, respectively. Under the optimized conditions, the time-course reactions of APPT production were performed with 1.0 mg ml^−1^ total PPT-type ginsenosides and 5.0 mg ml^−1^ DT-bgl for 6 h (Fig. 3b). After 4 h, APPT production reached a plateau. At this time, DT-bgl produced 0.40 mg ml^−1^ APPT, with a productivity of 100 mg l^−1^ h^−1^ and a molar conversion of 74.9%. The enzyme also produced 0.17 mg ml^−1^ R2 and 0.06 mg ml^−1^ Rg2 as by-products, with molar conversions of 18.8 and 6.3%, respectively.

### Determination of the added concentration of PF-bgl to DT-bgl for the complete conversion of ginsenoside R1 and glycosylated PPT-type ginsenosides in notoginseng root extract to APPT

**Fig. 4:**
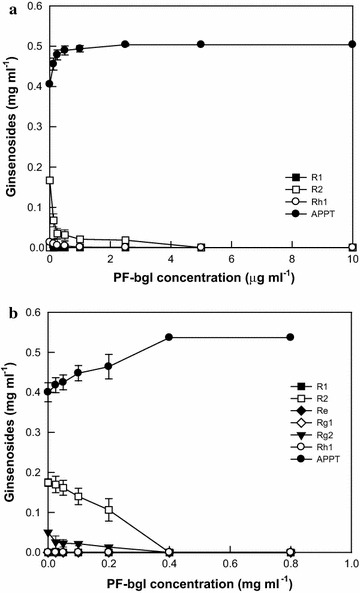
Determination of the minimal concentration of PF-bgl to DT-bgl for the complete conversion of PPT-type ginsenosides to APPT. **a** Ginsenoside R1. The reactions were performed at 80 °C in 50 mM citrate/phosphate buffer (pH 6.0) containing 1.0 mg ml^−1^ R1, 4.0 mg ml^−1^ DT-bgl, and PF-bgl for 6 h. **b** PPT-type ginsenosides in notoginseng root extract. The reactions were performed at 80 °C in 50 mM citrate/phosphate buffer (pH 6.0) containing 1.0 mg ml^−1^ total PPT-type ginsenosides, 5.0 mg ml^−1^ DT-bgl, and PF-bgl for 4 h. Data represent the means of three experiments, and error bars represent the standard deviation

The optimal pH and temperature values of DT-bgl and PF-bgl were reported to be 6.0 and 80 °C; and 5.5 and 95 °C, respectively (Lee et al. 2014; Oh et al. 2014). However, the activity of DT-bgl was completely abolished at pH 5.5 and 95 °C. Thus, all reactions were performed at pH 6.0 and 80 °C. DT-bgl at 4.0 mg ml^−1^ converted 1.0 mg ml^−1^ R1 to 0.40 mg ml^−1^ APPT with 0.17 mg ml^−1^ R2 and 0.02 mg ml^−1^ Rh1 as by-products for 6 h. To completely convert R2 and Rh1 to APPT, various concentrations of PF-bgl were added to the reaction solution. The residual concentration of R2 decreased with increasing PF-bgl concentration, and neither R2 nor Rh1 was detected at PF-bgl concentrations above 5 µg ml^−1^ (Fig. 4a). Therefore, 5 µg ml^−1^ was the optimal concentration of PF-bgl to completely produce APPT from R1 along with 4.0 mg ml^−1^ DT-bgl.

DT-bgl at 5.0 mg ml^−1^ converted 1.0 mg ml^−1^ total PPT-type ginsenosides in notoginseng root extract to 0.40 mg ml^−1^ APPT with 0.17 mg ml^−1^ R2 and 0.05 mg ml^−1^ Rg2 as by-products for 4 h. To completely convert R2 and Rg2 to APPT, various concentrations of PF-bgl were added to the reaction solution. The residual concentrations of R2 and Rg2 decreased with increasing PF-bgl concentration (Fig. 4b). R2 and Rg2 were not detected at concentrations above 0.4 mg ml^−1^ PF-bgl. Therefore, 0.4 mg ml^−1^ was the optimal concentration of PF-bgl along with 5.0 mg ml^−1^ DT-bgl for the complete production of APPT from glycosylated PPT ginsenoside in notoginseng root extract.

### Complete conversion of ginsenoside R1 and PPT-type ginsenosides in notoginseng root extract to APPT by DT-bgl combined with PF-bgl under the optimized conditions

**Fig. 5:**
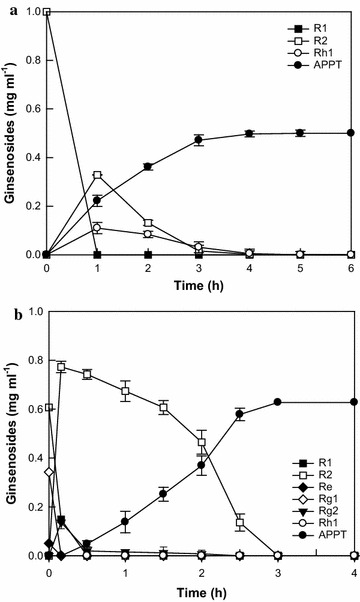
Time-course reactions for the biotransformations of PPT-type ginsenosides to APPT by DT-bgl combined with PF-bgl. **a** Ginsenoside R1. The reactions were performed at 80 °C in 50 mM citrate/phosphate buffer (pH 6.0) containing 1.0 mg ml^−1^ R1, 4.0 mg ml^−1^ DT-bgl, and 5 µg ml^−1^ PF-bgl for 6 h. **b** PPT-type ginsenosides in notoginseng root extract. The reactions were performed at 80 °C in 50 mM citrate/phosphate buffer (pH 6.0) containing 1.0 mg ml^−1^ total PPT-type ginsenosides, 5.0 mg ml^−1^ DT-bgl, and 0.4 mg ml^−1^ PF-bgl for 4 h. Data represent the means of three experiments, and error bars represent the standard deviation

The time-course reactions for the biotransformation of ginsenoside R1 to APPT were carried out for 6 h under the optimized conditions of pH 6.0, 80 °C, 1.0 mg ml^−1^ R1, 4.0 mg ml^−1^ DT-bgl, and 5 µg ml^−1^ PF-bgl. DT-bgl combined with PF-bgl converted 1.0 mg ml^−1^ R1 to 0.5 mg ml^−1^ APPT, with a productivity of 125 mg l^−1^ h^−1^ and a molar conversion of 100% after 4 h (Fig. 5a). The HPLC profiles at 0, 2, and 4 h are presented in Additional file 1: Figure S2.

The time-course reactions for the biotransformation of glycosylated PPT-type ginsenoside in notoginseng root extract to APPT were carried out for 4 h under the optimized conditions of pH 6.0, 80 °C, 1.0 mg ml^−1^ total PPT-type ginsenosides, 5.0 mg ml^−1^ DT-bgl, and 0.4 mg ml^−1^ PF-bgl. DT-bgl combined with PF-bgl converted 1.0 mg ml^−1^ total PPT-type ginsenosides in notoginseng root extract to 0.63 mg ml^−1^ APPT, with a productivity of 210 mg l^−1^ h^−1^ and a molar conversion of 100% after 3 h (Fig. 5b). The HPLC profiles at 0, 2, and 3 h are presented in Additional file 1: Figure S5.

### Complete conversion of ginsenosides F3 and F5 to APPT by DT-bgl combined with PF-bgl

Under the optimized conditions used for the complete conversion of ginsenoside R1 to APPT, the transformations of ginsenosides F3 and F5 to APPT were performed with 1.0 mg ml^−1^ of each ginsenoside by 4.0 mg ml^−1^ DT-bgl and 5 µg ml^−1^ PF-bgl for 8 h. DT-bgl combined with PF-bgl converted 1.0 mg ml^−1^ F3 and F5 to 0.62 mg ml^−1^ APPT for 4 and 6 h, with productivities of 155 and 103 mg l^−1^ h^−1^ and a molar conversion of 100%, respectively (Additional file 1: Figure S3).

## Discussion

The concentration ratios of PPT-type to PPD-type ginsenosides for Korean red ginseng, *P. quinquefolius* root, *P. quinquefolius* seed, *P. ginseng* root, and *P. notoginseng* root are 0.6, 1.0, 0.4, 1.3, and 1.4, respectively (Ko et al. 2008; Lee et al. 2014; Schlag and McIntosh 2006; Shin et al. 2015a). Thus, notoginseng root extract is the best substrate for APPT production based on the utilization efficiency of ginsenosides.

*Bacteroides* JY-6 (Bae et al. 2005) and β-glucosidases from *Acinosynnema mium* (Cui et al. 2013), *Aspergillus niger* (Liu et al. 2010), *Aspergillus* sp. 39 g (Wang et al. 2012), *Dictyoglomus turgidum* (Lee et al. 2014), *Gordonia terrae* (Shin et al. 2015b), *Penicillium aculeatum* (Lee et al. 2013), and *Penicillium decumbens* (Ko et al. 2003) have been reported to convert PPT-type ginsenosides as substrates to APPT. Crude β-glucosidase from *A. niger* converted ginsenoside Rf to APPT with a molar conversion of 90.4%. β-Glucosidases from *G. terrae* and *P. aculeatum* converted Rg1 and Rf to APPT with molar conversions of 65.8 and 90.0%, respectively. DT-bgl completely converted Rg1 and Rf to APPT, and converted R1 to APPT with a molar conversion of 75.6% (Lee et al. 2014). Therefore, the APPT-producing activity of DT-bgl using ginseng extract was reported to be the highest among the enzymes and cells tested (Lee et al. 2014) and DT-bgl was selected as the enzyme for APPT production in the present study. However, DT-bgl could not hydrolyze the outer rhamnose residue at C-6 in ginsenosides Re and Rg2 and the outer arabinopyranose residue at C-20 in ginsenoside F3 and showed low activity for the outer xylose residue at C-6 in ginsenoside R2 (Fig. 1). In contrast, PF-bgl hydrolyzed the outer rhamnose residue at C-6 in Re and Rg2 and the outer arabinopyranose residue at C-20 in ginsenosides F3, and the activity of PF-bgl for R2 was 19.3-fold higher than that of DT-bgl. These results suggest that all typical PPT ginsenosides can be efficiently converted to APPT by mutual complementation of the two enzymes (Fig. 2), and the two-enzyme system is expected to show the highest APPT productivity.

DT-bgl was reported to convert 1.0 mg ml^−1^ R1 to 0.36 mg ml^−1^ APPT with a productivity of 15 mg l^−1^ h^−1^ and molar conversion of 75.6%, and to convert 1.35 mg ml^−1^ PPT-type ginsenosides in notoginseng root extract to 0.62 mg ml^−1^ APPT with a productivity of 31 mg l^−1^ h^−1^ and a molar conversion of 81.2% (Lee et al. 2014). This is the only report of the production of APPT from R1 or PPT-type ginsenosides in ginseng root. In the present study, the reaction conditions of DT-bgl were optimized. Through the optimization, the productivity of APPT from R1 and notoginseng root extract increased by 4.4- and 3.2-fold, respectively (Fig. 3).

PF-bgl at 5 µg ml^−1^ for R1 and 0.4 mg ml^−1^ for notoginseng root extract as the minimal concentrations was added to 4.0 and 5.0 mg ml^−1^ DT-bgl, respectively. As a result, the two enzymes converted the complete amounts of R1 and glycosylated PPT ginsenosides in notoginseng root extract to APPT by complete hydrolysis of the by-products produced by DT-bgl for 8 h (Fig. 4). DT-bgl combined with PF-bgl completely converted R1 and converted 1.0 mg ml^−1^ total PPT-type glycosylated ginsenosides in notoginseng root extract to APPT, with productivities of 126 and 200 mg l^−1^ h^−1^, respectively (Fig. 5). The productivity and molar conversion of APPT from R1 and notoginseng root extract by DT-bgl combined with PF-bgl were 1.9-fold and 19%, and 2.0-fold and 25% higher than those obtained by DT-bgl alone under the optimized conditions, respectively, and were 8.4-fold and 24%, and 6.5-fold and 19% higher than previous results obtained with DT-bgl (Lee et al. 2014), respectively. Thus, DT-bgl combined with PF-bgl under the optimized conditions showed the highest productivity and conversion from R1 and ginseng extract ever reported.

DT-bgl has a weakness for APPT production because it could not hydrolyze Rg2 and F3 (Fig. 1) and could not convert Re to APPT (Fig. 2). In contrast, DT-bgl combined with PF-bgl completely converted all typical glycosylated PPT ginsenosides, including R1, R2, Re, Rg1, Rg2, Rh1, Rf, F1, F3, and F5, to APPT with molar conversion of 100% (Table 1). β-Glucosidase from *G. terrae* at 20 mg ml^−1^ converted 4.0 mg ml^−1^ Rg1 to 1.12 mg ml^−1^ APPT for 5 h, with volumetric and specific productivities of 224 mg l^−1^ h^−1^ and 11.2 mg g^−1^ h^−1^, respectively. DT-bgl converted 4.0 mg ml^−1^ Rg1 to 2.38 mg ml^−1^ APPT for 10 h, with volumetric and specific productivities of 297 mg l^−1^ h^−1^ and 74 mg g^−1^ h^−1^, respectively, which were 1.3- and 6.6-fold higher than those of β-glucosidase from *G. terrae*, respectively. β-Glucosidase from *P. aculeatum* at 5.7 mg ml^−1^ converted 0.5 mM Rf to 0.45 mM for 6 h, with a productivity of 35.8 mg l^−1^ h^−1^. The productivity of APPT using Rf by DT-bgl combined with PF-bgl was 297 mg l^−1^ h^−1^, which was 8.3-fold higher than that of β-glucosidase from *P. aculeatum*. Thus, DT-bgl combined with PF-bgl showed the highest activity for APPT production using all typical glycosylated PPT ginsenosides.

**Table 1:** Complete conversion of all typical PPT ginsenosides to APPT by DT-bgl combined with PF-bgl

Microorganism	Enzyme (mg ml^−1^)	Substrate (mg ml^−1^)	Pathway	Molar conversion (%)	Productivity (mg l^−1^ h^−1^)	References
*Aspergillus niger*	β-Glucosidase	Rf (1)	Rf → Rh1 → APPT	90.4	NC	Liu et al. (2010)
*Gordonia terrae*	β-Glucosidase (20)	Rg1 (4)	Rg1 → Rh1 → APPT	65.8	224	Shin et al. (2015b)
*Penicillium aculeatum*	β-Glucosidases (5.7)	Rf (1)	Rf → Rh1 → APPT	90.0	35.8	Lee et al. (2013)
*Dictyoglomus turgidum*	β-Glycosidase (1.3)	R1 (1)	R1 → R2 → Rh1 → APPT	75.6	15.0	Lee et al. (2014)
			Rg1 → Rh1 → APPT	100	92.3	
			Rf → Rh1 → APPT	100	92.3	
*Dictyoglomus turgidum* and *Pyrococcus furiosus*	β-Glycosidase (4) and β-Glycosidase (0.005)	R1 (1)	R1 → R2 and Rg1 → Rh1 → APPT	100	126	This study
		R2 (1)	R2 → Rh1 → APPT	100	303	
		Re (1)	Re → Rg1 and Rg2 → Rh1 → APPT	100	167	
		Rg1 (4,1)	Rg1 → Rh1 → APPT	100	297, 148	
		Rg2 (1)	Rg2 → Rh1 → APPT	100	202	
		Rh1 (1)	Rh1 → APPT	100	373	
		Rf (1)	Rf → Rh1 → APPT	100	297	
		F1 (1)	F1 → APPT	100	373	
		F3 (1)	F3 → F1 → APPT	100	155	
		F5 (1)	F5 → F1 → APPT	100	103	

*NC* not calculated

In conclusion, DT-bgl converted ginsenosides R1, R2, Rg1, Rf, Rh1 and F1 to APPT, but did not convert Re, Rg2, and F3 to APPT. PF-bgl converted Re and Rg2 to Rh1 and converted F3 to F1. Thus, DT-bgl combined with PF-bgl completely converted all typical glycosylated PPT ginsenosides, including R1, R2, Re, Rg1, Rg2, Rh1, Rf, F1, F3, and F5, to APPT. To the best of our knowledge, this is the first demonstration of the complete conversion of all typical glycosylated PPT ginsenosides to APPT and the highest concentration and productivity of APPT from ginseng extract reported to date. Moreover, this is the first report on the conversion of ginsenosides F3 and F5 to APPT. Therefore, DT-bgl combined with PF-bgl is an efficient biocatalyst for the production of APPT using ginseng extracts.

## Additional file

**Additional file 1: Figure S1.** Effects of enzyme and substrate concentrations on the production of APPT from ginsenoside R1 as a substrate by DT-bgl. **a** Effect of enzyme concentration. The reactions were performed at 80°C in 50 mM citrate/phosphate buffer (pH 6.0) containing R1 and 4.0 mg ml^−1^ DT-bgl for 1.5 h. **b** Effect of substrate concentration. The reactions were performed at 80°C in 50 mM citrate/phosphate buffer (pH 6.0) containing 1.0 mg ml^−1^ R1 and DT-bgl for 1.5 h. Data represent the means of three experiments, and *error bars* represent the standard deviation. Symbols: R1 (filled square), R2 (open square), Rh1 (open circle), APPT (filled circle). **Figure S2.** Effects of enzyme and substrate concentrations on the production of APPT from total PPT-type ginsenosides in notoginseng root extract as a substrate by DT-bgl. **a** Effects of enzyme concentration. The reactions were performed at 80°C in 50 mM citrate/phosphate buffer (pH 6.0) containing 1.0 mg ml^−1^ total PPT-type ginsenosides and 4.0 mg ml^−1^ DT-bgl for 5 h. **b** Effect of substrate concentration. The reactions were performed at 80°C in 50 mM citrate/phosphate buffer (pH 6.0) containing total PPT-type ginsenosides and 4.0 mg ml^−1^ DT-bgl for 5 h. Data represent the means of three experiments, and *error bars* represent the standard deviation. Symbols: R1 (filled square), R2 (open square), Re (filled diamond), Rg1 (open diamnod), Rg2 (filled triangle down), Rh1 (open circle), APPT (filled circle). **Figure S3.** HPLC profiles during the conversion of ginsenoside R1 to APPT using DT-bgl combined with PF-bgl. **a** 0 h. The ginsenoside R1 peak represents a single substrate. **b** 2 h. The ginsenoside R2, Rh1, and APPT peaks represent two intermediates and a product, respectively. **c** 4 h. The APPT peak represents a single product. **Figure S4.** HPLC profiles during the biotransformation of notoginseng root extract by DT-bgl combined with PF-bgl. **a** 0 h. Notoginseng was contained the R1, Re and Rg1 peaks. **b** 2 h. The ginsenoside Rg2 and APPT peaks represent intermediates and a product, respectively. **c** 3 h. The APPT peak represents a single product. **Figure S5.** Time-course reactions for the biotransformations of ginsenosides F3 and F5 as substrates to APPT by DT-bgl combined with PF-bgl. The reactions were carried out with 1.0 mg ml^−1^ F3 or F5, 4.0 mg ml^−1^ DT-bgl, and 5 µg ml^−1^ PF-bgl for 5 h. **a** Time-course reactions for the biotransformation of ginsenoside F3. **b** Time-course reactions for the biotransformation of ginsenoside F5. Data represent the means of three experiments, and *error bars* represent the standard deviation. Symbols: F3 (open triangle up), F5 (open triangle down), F1 (filled triangle up), APPT (filled circle).

## Abbreviations

APPT: 20(*S*)-protopanaxatiol
PPT: protopanaxatiol
DT-bgl: β-glycosidase from *Dictyoglomus turgidum*
PF-bgl: β-glycosidase from *Pyrococcus furiosus*
HPLC: high-performance liquid chromatography
