# Supplementary material for: Complete conversion of all typical glycosylated protopanaxatriol ginsenosides to aglycon protopanaxatriol by combined bacterial β-glycosidases
Source: AMB Express. 2018 Jan 24;8:8. doi: 10.1186/s13568-018-0543-1 (PMC5783978; doi:10.1186/s13568-018-0543-1)
Supplement: Supplementary file 1 — Additional file 1: Figure S1. Effects of enzyme and substrate concentrations on the production of APPT from ginsenoside R1 as a substrate by DT-bgl. a Effect of enzyme concentration. The reactions were performed at 80°C in 50 mM citrate/phosphate buffer (pH 6.0) containing R1 and 4.0 mg ml−1 DT-bgl for 1.5 h. b Effect of substrate concentration. The reactions were performed at 80°C in 50 mM citrate/phosphate buffer (pH 6.0) containing 1.0 mg ml−1 R1 and DT-bgl for 1.5 h. Data represent the means of three experiments, and error bars represent the standard deviation. Symbols: R1 (filled square), R2 (open square), Rh1 (open circle), APPT (filled circle). Figure S2. Effects of enzyme and substrate concentrations on the production of APPT from total PPT-type ginsenosides in notoginseng root extract as a substrate by DT-bgl. a Effects of enzyme concentration. The reactions were performed at 80°C in 50 mM citrate/phosphate buffer (pH 6.0) containing 1.0 mg ml−1 total PPT-type ginsenosides and 4.0 mg ml−1 DT-bgl for 5 h. b Effect of substrate concentration. The reactions were performed at 80°C in 50 mM citrate/phosphate buffer (pH 6.0) containing total PPT-type ginsenosides and 4.0 mg ml−1 DT-bgl for 5 h. Data represent the means of three experiments, and error bars represent the standard deviation. Symbols: R1 (filled square), R2 (open square), Re (filled diamond), Rg1 (open diamnod), Rg2 (filled triangle down), Rh1 (open circle), APPT (filled circle). Figure S3. HPLC profiles during the conversion of ginsenoside R1 to APPT using DT-bgl combined with PF-bgl. a 0 h. The ginsenoside R1 peak represents a single substrate. b 2 h. The ginsenoside R2, Rh1, and APPT peaks represent two intermediates and a product, respectively. c 4 h. The APPT peak represents a single product. Figure S4. HPLC profiles during the biotransformation of notoginseng root extract by DT-bgl combined with PF-bgl. a 0 h. Notoginseng was contained the R1, Re and Rg1 peaks. b 2 h. The ginsenoside Rg2 and AP [file 13568_2018_543_MOESM1_ESM.docx]

**Additional data**

**AMB Express**

Complete conversion of all typical glycosylated protopanaxatriol ginsenosides to aglycon protopanaxatriol by β-glycosidases from *Dictyoglomus turgidum* and *Pyrococcus furiosus*

Eun-Joo Yang , Tae-Hun Kim , Kyung-Chul Shin , Deok-Kun Oh

Corresponding author: D.-K. Oh

Department of Bioscience and Biotechnology, Konkuk University, Seoul 05029, Republic of Korea, E-mail: [deokkun@konkuk.ac.kr](mailto:deokkun@konkuk.ac.kr), Tel.: +82-2-454-3118; Fax: +82-2-444-5518

**a**

**b**

**Fig. S1** Effects of enzyme and substrate concentrations on the production of APPT from ginsenoside R1 as a substrate by DT-bgl. **a** Effect of enzyme concentration. The reactions were performed at 80°C in 50 mM citrate/phosphate buffer (pH 6.0) containing R1 and 4.0 mg ml^−1^ DT-bgl for 1.5 h. **b** Effect of substrate concentration. The reactions were performed at 80°C in 50 mM citrate/phosphate buffer (pH 6.0) containing 1.0 mg ml^−1^ R1 and DT-bgl for 1.5 h. Data represent the means of three experiments, and *error bars* represent the standard deviation.

**a**

**b**

**Fig. S2** Effects of enzyme and substrate concentrations on the production of APPT from total PPT-type ginsenosides in notoginseng root extract as a substrate by DT-bgl. **a** Effects of enzyme concentration. The reactions were performed at 80°C in 50 mM citrate/phosphate buffer (pH 6.0) containing 1.0 mg ml^−1^ total PPT-type ginsenosides and 4.0 mg ml^−1^ DT-bgl for 5 h. **b** Effect of substrate concentration. The reactions were performed at 80°C in 50 mM citrate/phosphate buffer (pH 6.0) containing total PPT-type ginsenosides and 4.0 mg ml^−1^ DT-bgl for 5 h. Data represent the means of three experiments, and *error bars* represent the standard deviation.

**a**


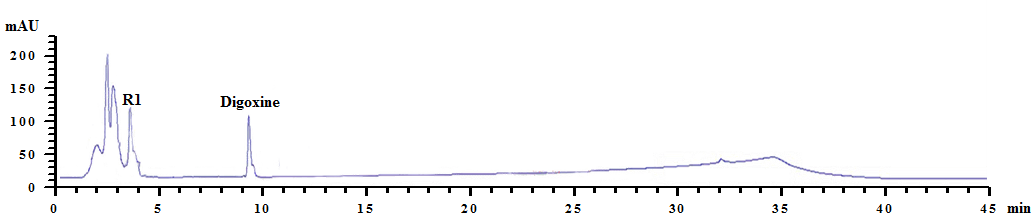


**b**


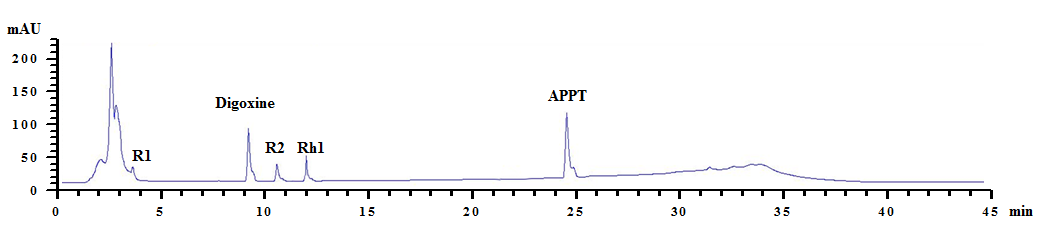


**c**


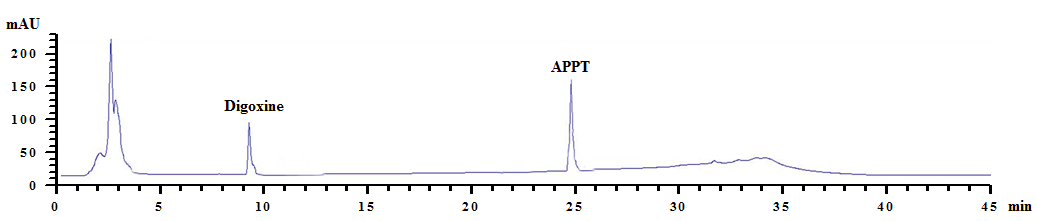


**Fig. S3**  HPLC profiles during the conversion of ginsenoside R1 to APPT using DT-bgl combined with PF-bgl. **a** 0 h. The ginsenoside R1 peak represents a single substrate. **b** 2 h. The ginsenoside R2, Rh1, and APPT peaks represent two intermediates and a product, respectively. **c** 4 h. The APPT peak represents a single product.

**a**


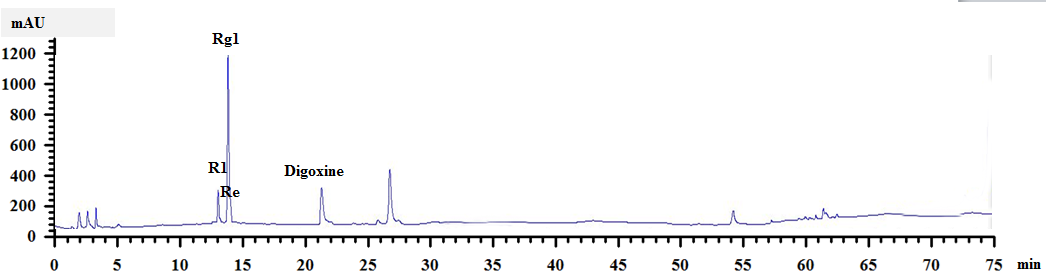


**b**


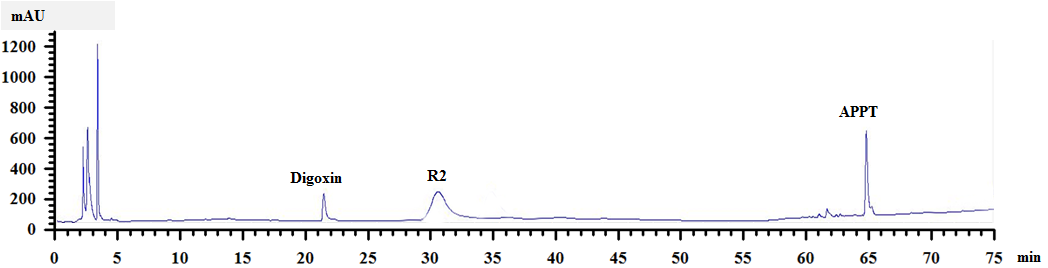


**c**


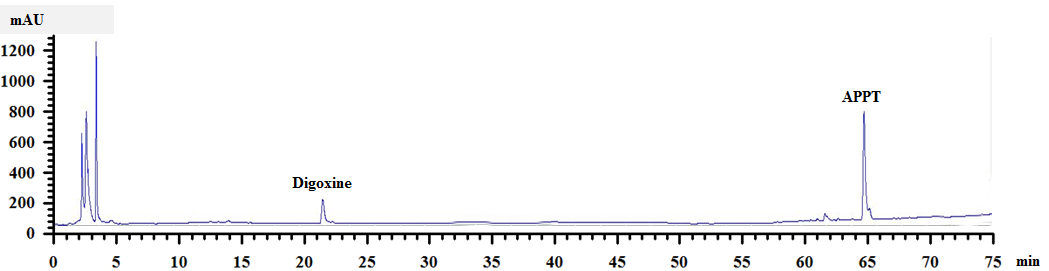


**Fig. S4** HPLC profiles during the biotransformation of notoginseng root extract by DT-bgl combined with PF-bgl. **a** 0 h. Notoginseng was contained the R1, Re and Rg1 peaks. **b** 2 h. The ginsenoside Rg2 and APPT peaks represent intermediates and a product, respectively. **c** 3 h. The APPT peak represents a single product.

**a**

**b**

**Fig. S5** Time-course reactions for the biotransformations of ginsenosides F3 and F5 as substrates to APPT by DT-bgl combined with PF-bgl. The reactions were carried out with 1.0 mg ml^−1^ F3 or F5, 4.0 mg ml^−1^ DT-bgl, and 5 µg ml^−1^ PF-bgl for 5 h. **a** Time-course reactions for the biotransformation of ginsenoside F3. **b** Time-course reactions for the biotransformation of ginsenoside F5. Data represent the means of three experiments, and *error bars* represent the standard deviation.
